# The Muscle Strength of the Knee Joint after ACL Reconstruction Depends on the Number and Frequency of Supervised Physiotherapy Visits

**DOI:** 10.3390/ijerph182010588

**Published:** 2021-10-09

**Authors:** Andrzej Czamara, Katarzyna Krzemińska, Wojciech Widuchowski, Szymon Lukasz Dragan

**Affiliations:** 1 Department of Physiotherapy, The College of Physiotherapy in Wroclaw, 50-038 Wroclaw, Poland; 2 Center of Rehabilitation and Medical Education in Wroclaw, 50-038 Wroclaw, Poland; katarzyna.krzeminskaa@gmail.com; 3 District Hospital of Orthopedics and Trauma Surgery, 41-940 Piekary Slaskie, Poland; sportmed@sportmed.com.pl; 4 Department of Regenerative and Restorative Medicine in Orthopaedics, Wroclaw Medical University, Borowska 213, 50-556 Wroclaw, Poland; s.dragan@umed.wroc.pl

**Keywords:** functional assessment, isokinetic torque, isometric torque, quadriceps and hamstring muscles

## Abstract

The aim of this study in anterior cruciate ligament reconstruction (ACLR) patients was to assess the effect of six months of supervised physiotherapy with a higher number of visits (SPHNV) compared to supervised physiotherapy with a lower number of visits (SPLNV) on the maximal peak torque (PT) and isometric torque (IT) of values obtained for hamstring (H) and quadriceps (Q) muscles of the knee joints under isokinetic and isometric conditions. Hypothesis: SPHNV improves IT and PT more than SPLNV. Group I had ACLR with a higher number of visits (*n* = 20), Group II had ACLR with a lower number of visits (*n* = 20), and Group III served as the control (*n* = 20). In Groups I and II, IT values were measured for quadriceps and hamstring muscles of the knee joints in the 13th and 24th weeks and for PT in the 18th and 24th weeks after ACLR (60 and 180 °/s). In group III, the measurements were taken once. The isometric torque and isokinetic peak torque values were measured in N*m and they were normalized to body mass as relative IT (RIT) and relative PT (RPT) were expressed in N*m/kg. Results: In both ACLR groups, the RIT and RPT values obtained from the operated knee joints significantly increased in the 24 weeks following ACLR compared to the uninvolved side. Group II had significantly lower RIT and RPT values for quadriceps and hamstring muscles of the operated limbs compared with the uninvolved limbs (*p* = 0.008, *p* = 0.001). In group I, the larger number of visits positively correlated with the higher PT for quadriceps and hamstring muscles of the operated and uninvolved knees (from *r* = 0.506; *p* = 0.023 too *r* = 0.566; *p* = 0.009), respectively. Six months of SPHNV positively correlated with and improved the IT and PT values in patients after ACLR much more significantly than six months of SPLNV.

## 1. Introduction

Anterior cruciate ligament (ACL) injuries account for up to 60% of all knee injuries in dynamic and pivoting sports [1]. Statistically, for every 100,000 people, isolated ACL tears occur in 70 knees [2] This problem is important for public health policy because it often concerns the treatment of people who are professionally and physically active, which is related to health, economic, and social aspects. Inadequate orthopedic and physiotherapeutic treatment promotes the occurrence of re-injuries of the knee joints, which in turn increases the costs and time of treatment, reduces its effectiveness, causes absenteeism in sports and professional work, and, in the long term, contributes to the earlier occurrence of osteoarthritis, a common disease in the population [3,4].

At present, there is no definitive answer to the question of the effect of the number and frequency of patient visits in supervised physiotherapy on the biomechanical parameters of muscles of the knee joints after ACLR, because previous studies have used different research methodologies as well as different physiotherapy protocols and surgical techniques [5,6,7,8,9]. A meta-analysis performed by Papalia et al. (2013) concluded that additional research is necessary for the accurate assessment of home, supervised, and ambulatory rehabilitation on knee joint function in patients after knee surgery [10].

Supervised postoperative physiotherapy after ACLR is carried out by a physiotherapist in an outpatient rehabilitation center based on a detailed protocol, which is agreed on with the physician [11]. One visit lasts about 2 h, in direct contact with a physiotherapist [12,13,14]. In addition, depending on the stage of postoperative physiotherapy, the patient has to perform exercises recommended by a physiotherapist at home. The patients who want to return to training and next to return to dynamic sport the supervised physiotherapy (SP) should continue for at least six or more months [13,14]. The final decision to return the patient to sports activity should be based on a clinical examination, an assessment of the restored functions of the joint and the whole body, and the patient’s mental readiness [15].

Czuppon et al. (2014) suggested that variables associated with a return to sports after ACLR included higher quadriceps strength, less effusion, less pain, greater tibial rotation, higher activity score, higher athletic confidence, higher preoperative knee self-efficacy, lower kinesiophobia, and higher preoperative self-motivation [16]. Wilk et al. (2017) found that current rehabilitation programs focus on strengthening exercises and proprioceptive and neuromuscular control [17]. Restoration of muscle strength is one of six criteria for the return to sports after anterior cruciate ligament reconstruction and, in addition, hamstring to quadriceps strength ratio deficits were associated with an increased risk of ACL graft rupture [5]. De Carlo et al. (1997) and Grant et al. (2005) did not observe any significantly higher peak torque (PT) values of the knee muscles between the groups with supervised physiotherapy and home physiotherapy after ACLR [9,18]. Królikowska et al. (2018) have shown that carrying out 6 months or more of regular postoperative physiotherapy supervision, compared to physiotherapy carried out for less than 6 months after ACLR, was more effective for improving the peak torque, power, and work of the knee muscles, as well as speed running and patients’ agility [13,14]. Hsiao et al. (2014) showed, 6 months after ACLR, that the quadriceps of the injured knees were 50% weaker in both isometric and isokinetic conditions because none of the patients had received regular rehabilitation [19]. However, Sousa et al. (2017) demonstrated that patients with excellent performance regarding their isokinetic strength and functional testing at six months after ACL reconstruction have superior knee function and higher activity levels at midterm follow-up [20].

However, the cited studies did not conclude on the effect of the different number and frequency of supervised physiotherapy visits, performed by the physiotherapist according to one standardized physiotherapy protocol, on the maximum peak torque values and maximum isometric torque values obtained for the quadriceps and hamstring muscles of the knee joints after a 6-month physiotherapy program for patients after ACLR. Conducting such preliminary studies may open up a discussion on determining the scope of postoperative physiotherapy needed to restore muscle strength as one of several important criteria for patients’ returning to physical activity or professional work after ACLR, and for determining the optimal number of physiotherapeutic visits, which may be relevant to the final cost of treatment.

The aim of this study is to assess the effect of 60 or more supervised physiotherapy visits compared to fewer than 60 supervised physiotherapy visits, performed and supervised by a physiotherapist for 24 weeks in patients who received ACLR on the maximal peak torque and maximal isometric torque values obtained for the hamstring and quadriceps muscles of the knee joints.

It was hypothesized that a higher number and frequency of supervised physiotherapy visits six months after ACLR positively influences the relative isometric and peak torque values obtained for hamstring and quadriceps muscles of the knee joints compared to a lower number and frequency of supervised physiotherapy visits.

## 2. Materials and Methods

The study was conducted according to the ethics and principles of Declaration of Helsinki. The study was approved by The Scientific Research Ethics Commission of the College of Physiotherapy in Wroclaw, Poland 2/2012 (18 November 2012) and by The Scientific Research Ethics Commission of Academy of Physical Education in Wroclaw, Poland 2006 (18 May 2006).

The sample size was estimated on the basis of 10 randomly selected results at the design stage of the study. Means and standard deviations of the results of relative isometric torque (RIT) obtained under isometric tension (0 °/s) in 13 weeks and 24 weeks in patients after ACLR were used to analyze the estimation of sample size (the estimated sample size for a two-sample paired-means test–paired *t*-test). Parameters: 13 weeks—mean x = 2.67 N*m/kg, standard deviation SD = 0.9 N*m/kg; 24 weeks—x = 3.51 N*m/kg, SD = 0.95 N*m/kg; the alpha level was set at 0.05, and the power of the test was set at 0.8. No correlation of evaluated variables was assumed and a two-sided null hypothesis was adopted. On the basis of the parameters, an estimated sample size equal to 20 participants in each group was obtained. The estimation of sample size was performed using Statistica 13 (TIBCO Software Inc., Palo Alto, CA, USA).

The initial sample consisted of 105 patients (15 females and 90 males) who decided to start the postoperative physiotherapeutic procedure after ACLR in the rehabilitation center, where the study was conducted. All the patients that participated in the study were informed of the purpose and approach to be used and they all signed an informed consent form prior to starting the postoperative physiotherapeutic procedure. All participants (the patients and the participants from the control group) included in the study were informed of the purpose and approach to be used, and they all signed an informed consent form to participate in the study.

**Figure 1:**
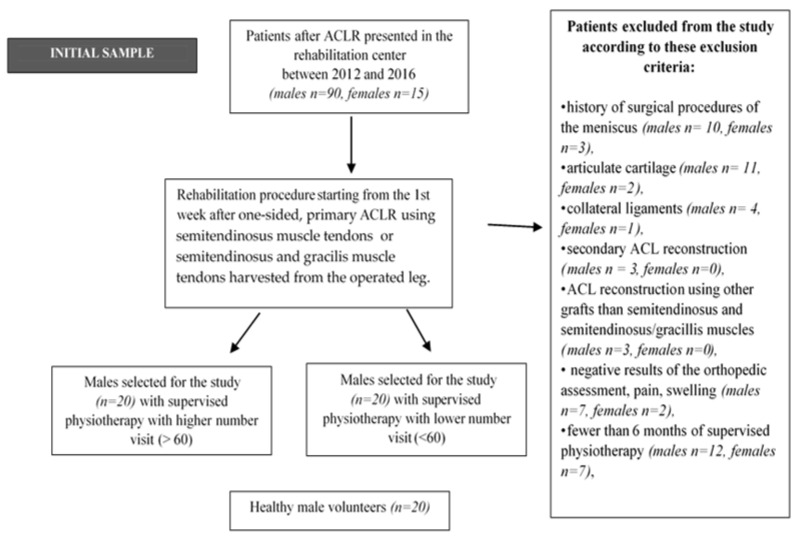
Flowchart of the study.

Based on the inclusion criteria (Figure 1), 40 males were eligible for the study after ACLR. Moreover, qualification for the two experimental ACLR groups was based on the number of visits during a 24-week, four-stage physiotherapy, where patients with 60 or more supervised physiotherapy visits were assigned to ACLR group I (*n* = 20, *x* = 74.1 SPHNV). Patients who participated in fewer than 60 supervised physiotherapy visits were assigned to ACLR group II (*n* = 20, *x* = 31.7 SPLNV). The average frequency of weekly supervised physiotherapy visits during the 6-month program was 3.13 and 1.32 in groups I after ACLR and II after ACLR, respectively. In both groups, the time of supervised physiotherapy was 2 h a day, with an experienced physiotherapist. Moreover, the patients obtained recommendations from the physiotherapist to perform and continue exercises at home. The patients from both ACLR groups were motivated by the physiotherapist to undergo systematic postoperative physiotherapy. The patients themselves decided on the number of visits they participated in during the 6-month physiotherapy. The therapist did not influence their decisions. Group III, which was the control group, included males with no knee joint injuries (*n* = 20).

The groups were uniform in terms of age, body mass, and body height (Table 1). The level of physical activity in all three groups was comparable at 6–7 according to the Tegner Activity Scale [21]. Patients from group I and group II regularly participated in various sports (football, volleyball, skiing, basketball) before ACL injury, and they declared that, after the end of treatment, they would like to return to physical activity. These studies were purely retrospective and observational studies.

### 2.1. Surgical Procedure

The patients from group I and group II underwent post-traumatic primary unilateral one-incision arthroscopically ACLR with the use of autologous ipsilateral semitendinosus or semitendinosus and gracilis muscle tendons graft taken from the operated leg. The time between the injury and ACLR in the studied groups of patients did not exceed 3 months. The distribution of the operated dominant lower legs and types of graft were comparable between groups I and II (Table 2), thus reducing the impact of these two types of graft on possible partial different histological conditions of the healing process, possible differences in the torque of the flexor muscles’ values, or differences in range of motion. However, a review of the literature indicates a gradual progress in the regeneration of these tendons and an improvement in muscle strength [22,23,24].

A recent comprehensive literature review underlines that firm tendon-to-bone healing was not always necessary for clinical stability of the knee joint. The underlying graft bone healing process is far from understood in human ACL reconstruction with hamstring muscles [25].

### 2.2. Physiotherapeutic Procedure

Group I and group II patients after ACLR participated in the four-stage physiotherapy program in the Center of Rehabilitation (Table 3) after ACLR, lasting 24 weeks, based on a previously published protocol [11] with modifications [6,7] that are briefly described in this paper [6,7,11].

### 2.3. Measurement Procedures

All the study participants underwent measurements of knee extensor and flexor muscle torque under isokinetic conditions using the Humac Norm Testing & Rehabilitation System (CSMI Computer Sports Medicine, Inc., Stoughton, MA, USA) [26] and under isometric conditions (SUMER, Poland) [11]. All the measurements were performed by the same examiner. The isometric torque (IT) values were taken under conditions of maximal isometric tension, while maximal peak torque (PT) values during the isokinetic test were measured in N*m for extensor and flexor muscles of the knee joints in the Department of Physiotherapy of the College of Physiotherapy in Wroclaw. In group I and group II, the isometric torque values were measured in the 13th and 24th weeks after ACLR and for peak torque of isokinetic conditioning in the 18th and 24th weeks after ACLR (for 60 and 180 °/s). In group III, the measurements were taken once. The isometric torque and isokinetic peak torque values were normalized to body mass as relative IT (RIT) and relative PT (RPT) and were expressed in N*m/kg. In the studied groups, biomechanical measurements were preceded by a 12 min warm-up. IT values were measured using the UPR-1 measuring system with the Moment 2 computer program (SUMER, Poland). IT values were measured in knee joint extensor muscles in the supine position at 70° of the knee flexion and knee joint flexors in the prone position at 30° of the knee flexion with the maintained stability of the pelvic area and the thigh of the tested leg. A detailed research methodology is presented in an earlier publication [11,26]. On the same day, after a 5 min break, PT values were measured in the extensor and flexor muscles of the knee joint under isokinetic conditions in seated patients using the Humac Norm^TM^ Testing & Rehabilitation System (CSMI Computer Sports Medicine, Inc., Stoughton, MA, USA). Two angular velocities were applied, namely 60 °/s (5 repetitions) and 180 °/s (10 repetitions), with a 2 min interval between the series. The detailed measurement methodology was based on a separate publication [26]. The length of the lever arm was 42 cm in both types of measurement. In the control group, the values were first measured in the dominant leg and then measured in the non-dominant leg. In the two ACLR groups, the measurement was first taken in the uninvolved leg, and then in the operated leg. Measurements of peak torque of the knee extensor and flexor muscles, carried out on the Humac Norm ^TM^ Testing & Rehabilitation System during isokinetic tests, showed that intraclass correlation coefficients (ICCs) ranged from 0.74 to 0.89 and, for internal and external shin rotator muscles in the knee joints, ICC ranged from 0.95 to 0.99 [27,28].

### 2.4. Statistical Analysis

The results were subjected to statistical analysis using Microsoft Office Excel 2016 and IBM SPSS Statistic for Windows, Version 20.0 (IBM Corp., Armonk, NY, USA). Torque values were expressed in kg of body mass to obtain relative torque values for both tests (N*m/kg). The number of individuals was indicated as *n*. At the beginning of the analysis, arithmetic mean values (*x*) and standard deviations (SD) for the description of variables were calculated. Shapiro–Wilk’s test was used to verify the distribution normality of the studied variables.

A comparison of the results of relative isometric torque (RIT) obtained under isometric tension (0 °/s) and relative peak torque (RPT) in the isokinetic tests (60 °/s and 180 °/s) values between first and second measurement was carried out using paired two-sample *t*-test. A comparison of the results between the operated leg and uninvolved leg or between the dominant leg and non-dominant leg in each group was carried out using an unpaired two-sample *t*-test. In cases where inter-group and intra-group comparisons were made, the Bonferroni correction was applied.

Inter-group comparisons of age, body mass, and body height were performed using the one-way ANOVA test and Tukey’s post hoc test (significance level was set at *p* < 0.05). The comparison of RIT and RPT results between groups, taking the first and second measurement into account, was performed using repeated-measures ANOVA with post-hoc test (Honest Significant Difference test).

Pearson’s linear correlation coefficient (*r*) was calculated for the force and direction of linear correlation between the number of supervised postoperative physiotherapy visits, and the isometric and relative peak torque in group I (SPHNV *n* = 20) and group II (SPLNV *n* = 20) The values corresponding to all two-dimensional associations were classified as negligible (0.00–0.30), low (0.31–0.50), moderate (0.51–0.70), high (0.71–0.90), and very high (0.901–1.00) [29]. The statistical significance level was set at *p* < 0.05.

## 3. Results

During the first measurement, in groups I and II, the relative isometric torque (RIT) and the relative peak torque (RPT) of extensor (Q) and flexor (H) muscles in the operated knee joints were significantly lower (*p* ≤ 0.001) than those of those on the uninvolved side (Table 4). Between the first and the second measurements, a statistically significant increase in RIT and RPT values was noted (*p* < 0.008 to *p* ≤ 0.001) in both studied groups. In group I, in the twenty-fourth week after ACLR (second measurement) in five of six cases, no significant differences in RIT and RPT values were noted between the muscle groups of the operated knee joints and the uninvolved side. In group II (twenty-fourth week following ACLR), in each of the six studied biomechanical tests, the RIT and RPT values obtained from the operated side were significantly lower for both studied muscle groups compared with the uninvolved side (*p* < 0.008 do *p* ≤ 0.001), as presented in Table 4. In group III (control) in five of six cases, no significant differences in RIT and RPT values were noted between the muscle groups of the right and the left knee joints (Table 4).

In the assessment of RIT and RPT measurements for extensor muscle, statistically significant differences were observed, accounting for division into groups or measurement time, as well as group and time interaction (main effect: *p* < 0.05). Post hoc analysis showed that significant differences were observed between groups I and III and II and III were observed in the first measurement. There were no statistically significant differences between the groups in the second measurement. Moreover, in groups I and II, statistically significant increases in the results between the first and second measurement were observed (Table 5).

In the assessment of RIT measurement for flexor muscle, statistically significant differences were observed, accounting for division into groups or measurement time, as well as the interaction between the group and time. In the case of RPT measurements, statistically significant differences were found, taking into account the differences between the first and second measurement (time), and in the case of the measurement of 180 deg/s, the main effect of *p* < 0.05 was observed for the interaction of time and group. Post hoc analysis showed that significant differences were observed between groups I and III and II and III, accounting for only the first measurement in the RIT assessment. Moreover, in groups I and II, statistically significant increases were observed between the first and second measurement (Table 5).

It was observed that almost all the differences obtained between the first and second measurement differ between groups. No significant differences were found in the measurement of RPT of 60 °/s for flexors. Post-hoc analysis showed no statistical significance in the comparison between groups II and III, accounting for the differences between the first and second measurement of RPT of 60 °/s for extensors and of RIT for flexors (Table 5)

In group I, a statistically significant and moderately positive Pearson’s linear correlation coefficient (*r*) was found between a with a higher number of supervised postoperative physiotherapy visits and a higher relative peak torque (RPT) for extensor muscles in operated and uninvolved knee joints in the isokinetic tests (60 °/s and 180 °/s) and for flexor muscles of the operated and uninvolved knee joints in the isokinetic tests (180 °/s), which is presented in Table 6. In group II, no statistically significant differences and a negligible low Pearson’s linear correlation coefficient (*r*) were found between the patients who participated in supervised physiotherapy with a lower number of visits, which is presented in Table 6.

## 4. Discussion

The study aimed to assess the effect of 60 or more supervised physiotherapy visits compared to fewer than 60 supervised physiotherapy visits, performed and supervised by a physiotherapist over the 24 weeks after ACLR on patients’ maximal peak torque and maximal isometric torque values for hamstring and quadriceps knee joint muscles.

Our research has shown that the hypothesis that a higher number and frequency of supervised physiotherapy visits carried out six months after ACLR positively influences the relative isometric and peak torque values obtained for hamstring and quadriceps knee joint muscles, compared to the lower number and lower frequency of supervised physiotherapy visits, has been confirmed. These values became more similar to those obtained from the uninvolved side compared with the results obtained from group II, who underwent SPLNV. In addition, in group I, between the first measurement and the last, the dynamics of the increase in the value of moments of strength of the studied muscle groups was higher compared to group II. There was also a significant correlation between a higher number and frequency of supervised physiotherapy visits and higher values of moments of strength for the studied muscle groups in operated knee joints.

However, due to the small number of participants in the study groups, the results of our research should be treated as pilot studies.

It can be assumed that our research will provoke other researchers to further study and contribute to the broadening knowledge on the impact of the established protocol of six months of conducted and supervised physiotherapy with more supervised physiotherapeutic visits compared to fewer supervised physiotherapy visits with one experienced physiotherapist after ACLR, under the very strict repetitive conditions of postoperative treatment and conducted research.

Pua et al. (2017) conducted an assessment of the impact of a different number of rehabilitation visits between patients who did and did not participate, and assessed the fitness of these patients after 6 months using only the English and Chinese SF 36 health survey scales. However, they did not conduct biomechanical studies. They found that patients who had more visits (two or more) and more frequently participated in physiotherapy programs after knee joint surgery obtained better treatment results in the English and Chinese SF 36 Health Survey Scales compared with patients who had fewer visits (only one) and less frequently participated in physiotherapy programs. Still, they had better results compared with patients who did not participate in any physiotherapy program [30].

Han et al. (2015) found that, in their group of patients after ACLR, supervised physiotherapy with a higher number of visits (SPHNV) and greater frequency of visits were associated with an earlier return to recreational physical activity, compared with the results for patients who participated in supervised physiotherapy with a lower number of visits (SPLNV) [31]. Artz et al. (2015) and Vervest et al. (1999) indicated a moderately better effect of supervised physiotherapy; however, this issue requires further research [32,33].

Grant et al. (2010) indicated a better effect of home-based rehabilitation compared with supervised rehabilitation [8]. Some authors also indicated the equal relevance of difficulty with a complete assessment of supervised physiotherapy compared with other types of physiotherapy regarding its scope, home physiotherapy, the number of visits, and the length of sessions [34,35,36,37,38,39,40,41,42,43,44]. Moreover, the researchers applied different approaches at different times following the surgery [10]. Palmieri-Smith et al. (2015) noted that patients with low quadriceps strength displayed greater movement asymmetries at the knee in the sagittal plane. According to the authors, in their sample, quadriceps strength was related to movement asymmetries and functional performance. Rehabilitation following ACL reconstruction needs to focus on maximizing quadriceps strength, which will likely lead to more symmetrical knee biomechanics [45]. Ericsson et al. (2013) showed that the majority of active young patients regain physical performance and muscle strength after a structured exercise program. On the other hand, a poor physical performance at the end of rehabilitation was predictive of worse patient-reported outcomes at 2 and 5 years, regardless of treatment [46]. Other authors indicate the necessity of restoring the strength of knee joint flexor muscles where grafts were taken after ACLR, as it is necessary to restore a proper muscular balance, namely, the so-called biomechanical-flexor-to-extensor ratio of peak torque values in the operated knee joints [6,47]. Restoring and increasing quadriceps strength is essential to maximizing the functional ability of the operated knee joint [48]. Moreover, restoring biomechanical parameters and neuromuscular coordination between the extensor and flexor muscles of the operated knee joint can decrease the value of shear forces affecting the anterior cruciate ligament (ACL), particularly within the range of motion in the last tens of degrees of extension in the open kinematic chain [6,49]. There are few studies on this issue reported in the literature, and the existing studies have not monitored the effectiveness of individual stages of supervised multi-stage physiotherapy for patients after ACLR, or described the detailed therapeutic procedures, individualization, and duration of every single visit [6,34,35,36,37,38,39,40,41,42,43,44,50,51].

Our research presents the physiotherapy protocol described in the literature that was previously subject to various studies [11,52].

The strengths of this study include the one-physiotherapeutic-protocol procedure, conducted and monitored by the same experienced physiotherapist and physicians at the same rehabilitation center, using the same therapeutic and testing equipment. All the procedures from surgery to physiotherapy were carried out in patients,” day after day”, in highly repeatable conditions to obtain the most critical evaluation and analysis of the biomechanical function of the knee joint following complex surgical treatment combined with physiotherapy.

Our study has some limitations resulting from the application of measurements and the analysis of biomechanical parameters in one plane of motion in the knee joint [6]. In addition, many functional tests were applied to assess different types of locomotion and whole-body fitness combined with other biomechanical tests and clinical assessments of patients [12,53,54].

Another limitation is the lack of comparison of our study outcome with the results obtained from a sample of females after ACLR. Such a comparison could answer to the question of whether dimorphism has any influence on the study results. Moreover, earlier studies were conducted and, in the future, it will be necessary to continue the distant assessment of treatment effects [27]. It is also recommended that this study be conducted in patients after ACLR using different grafts, e.g., artificial grafts or grafts harvested from dead bodies or other areas of the patient’s body. Other surgical techniques and graft fixation methods, as well as the regeneration of the ACL graft, should also be considered [22].

The main findings of the study provide practical information for sports medicine doctors, physiotherapist, coaches, and athletes in terms of postoperative physiotherapy after ACLR.

## 5. Conclusions

Our research has confirmed the hypothesis that a six-month supervised physiotherapy program applied to patients after ACLR, with 60 or more visits and more frequent participation, resulted in significantly better recovery of relative isometric and peak torque values of extensor and flexor muscles in operated knee joints as compared to patients after ACLR, with fewer confirmed supervised physiotherapy visits.

For patients after ACLR who would like to return to physical activity, the implementation of an average of 75 supervised physiotherapy visits is recommended. This number of visits allows patients to gain strength in the extensor and flexor muscles, similar to those obtained in the uninvolved knee joints and the control group results.

## Figures and Tables

**Table 1:** Characteristics of the study groups.

	Age (years)		Body Mass (kg)		Body High (cm)	
	*x*	SD	*x*	SD	*x*	SD
Group I (*n* = 20)	27.80	5.48	82.75	9.00	183.60	7.83
Group II (*n* = 20)	25.10	6.58	80.05	8.98	181.45	6.13
Group III (*n* = 20)	24.85	3.12	82.95	9.16	181.85	5.45
*p*	0.154		0.530		0.549	

SD: standard deviation; *x*: arithmetic mean; *p*: statistical significance level; Group I: patients who regularly participated in supervised physiotherapy with a greater number of visits (SPMNV); Group II: patients who participated in supervised physiotherapy with a fewer number of visits (SPFNV); Group III: control group.

**Table 2:** Characteristics of dominant leg in groups I, II, and III and operated legs and type of graft in groups I and II.

**TESTING GROUP**	**DOMINANT LEG**		**OPERATED LEG**		**GRAFTS**	
	Right	Left	Dominant	Non-Dominant	Semitendinosus	Semitendinosus & Gracilis
Group I (*n* = 20)	11	9	16	4	12	8
Group II (*n* = 20)	12	8	17	3	14	6
Group III (*n* = 20)	19	1	-	-	-	-

**Table 3:** Characteristics of the four-stage physiotherapy program in the Center of Rehabilitation and Medical Education for patients after ACLR.

**Stage I** (1th–5th week postoperatively)	Cool packs, passive movement on a CPM device, mobilization of the patellofemoral joint, soft tissues in the knee joint area and musculo-fascial muscular and fascial groups; electrostimulation of the vastus medialis of the quadriceps muscle was applied, followed by cryotherapy, isometric exercises with hand-controlled muscle resistance beyond the operated area, exercises of the uninvolved leg, upper limbs and trunk muscles; proprioception exercises were performed in closed kinematic chains with controlled pressure generated by the operated limb on the tensometric platform. Gait was trained with crutches, and exercises were gradually introduced, including balancing exercises and movement coordination exercises, and gait training without crutches was introduced at the end of the stage.
**Stage II** (6th–12th week postoperatively)	Continue stage I; exercises on a cycle ergometer and gait training technique performed on a hard surface, treadmill, unstable surface and stairs. The level of exercise difficulty was increased. Semi-squats were performed with both legs and gradually with one leg, body stability training. Concentric-eccentric exercises were introduced for the operated leg in the sagittal plane, exercises of other large muscle groups of individual body parts (except knee joint extension with resistance in an open kinematic chain).
**Stage III** (13th–20th week postoperatively)	Continue stage II; based on biomechanical measurements, supervised and monitored isometric training with gradual resistance for the quadriceps and flexor muscles was introduced for the operated leg under static conditions. After orthopedic examination and biomechanical tests, individual separate trot parameters allowed for running and jumping, and the elements of plyometric training were introduced with an emphasis on landing techniques. From the 18th week following ACLR, individual strength training was started under isokinetic conditions for extensor and flexor muscles of the knee joint in a limited range of motion, and the elements of the techniques of occupational activity-specific movements were introduced individually for each patient.
**Stage IV** (21st week postoperatively to 6th–8th month postoperatively)	Continue stage III; exercises specific for a given sports discipline and occupational activities were performed. Proper specific movement skills coordination was gradually practiced. Running speed was increased on a treadmill, and running with a sudden change of direction was introduced. Movement coordination training was applied, as well as neuromuscular training. Training of lower and upper body strength, velocity, jumping ability and agility, dynamic proprioception and physical fitness and exercises were introduced to prepare the patient for the return to play. Plyometric exercises, running at maximum speed and changing movement directions, and special exercises aimed at improving power and speed were applied. Between sessions: massage or low-intensity swimming pool activities; recovery protocols.

**Table 4:** Relative isometric torque (RIT) obtained under isometric tension (0 °/s) between 13 and 24 weeks after ACLR and relative peak torque (RPT) in the isokinetic test (60 °/s and 180 °/s) values between 18 and 24 weeks after ACLR for flexor and extensor muscles of the knee joints in groups I and II; for group III, these measurements were taken once.

RIT and RPT (N*m/kg) in Group I after ACLR (SPHNV)							
**Angular Velocity**	**Week after ACLR**	**Knee Joint Extensors**		*p* **	**Knee Joint Flexors**		*p* **
		**Operated Leg** ***x* ± SD**	**Uninvolved Leg** ***x* ± SD**		**Operated Leg** ***x* ± SD**	**Uninvolved Leg** ***x* ± SD**	
0 °/s	13	2.36 ± 0.79	3.41 ± 0.66	≤0.001	1.09 ± 0.36	1.35 ± 0.39	≤0.001
	24	3.72 ± 0.95	3.86 ± 0.97	0.556	1.51 ± 0.38	1.63 ± 0.33	0.396
*p* *		≤0.001	1.00	*p* *	≤0.001	0.080	
60 °/s	18	1.97 ± 0.44	2.68 ± 0.43	≤0.001	1.40 ± 0.30	1.58 ± 0.23	0.001
	24	2.56 ± 0.56	2.71 ± 0.48	0.320	1.55 ± 0.30	1.65 ± 0.25	0.200
*p* *		≤0.001	1.00	*p* *	0.428	1.00	
180 °/s	18	1.39 ± 0.37	1.89 ± 0.21	≤0.001	0.98 ± 0.26	1.13 ± 0.21	0.012
	24	1.81 ± 0.40	2.06 ± 0.31	≤0.001	1.16 ± 0.24	1.24 ± 0.21	0.092
*p* *		≤0.001	0.172	*p* *	0.100	0.492	
RIT and RPT (N*m/kg) in Group II after ACLR (SPLNV)							
**Angular Velocity**	**Week after ACLR**	**Knee Joint Extensors**		*p* **	**Knee Joint Flexors**		*p* **
		**Operated Leg**	**Uninvolved Leg**		**Operated Leg**	**Uninvolved Leg**	
0 °/s	13	1.95 ± 0.75	3.56 ± 0.42	≤0.001	1.07 ± 0.21	1.45 ± 0.25	≤0.001
	24	3.19 ± 0.78	3.85 ± 0.78	≤0.001	1.40 ± 0.42	1.61 ± 0.31	0.024
*p* *		≤0.001	0.604	*p* *	0.012	0.292	
60 °/s	18	1.82 ± 0.41	2.84 ± 0.43	≤0.001	1.36 ± 0.31	1.63 ± 0.27	≤0.001
	24	2.25 ± 0.44	2.74 ± 0.44	≤0.001	1.51 ± 0.35	1.69 ± 0.30	0.004
*p* *		0.003	1.00	*p* *	0.676	1.00	
180 °/s	18	1.34 ± 0.28	1.99 ± 0.17	≤0.001	0.98 ± 0.23	1.15 ± 0.21	≤0.001
	24	1.65 ± 0.31	2.06 ± 0.27	≤0.001	1.13 ± 0.26	1.24 ± 0.20	0.032
*p* *		0.008	1.00	*p* *	0.256	0.632	
RIT and RPT (N*m/kg bm) in GROUP III (CONTROL)							
**Angular Velocity**		**Knee Joint Extensors**		*p* ***	**Knee Joint Flexors**		*p* ***
		**Dominant Leg**	**Non-Dominant Leg**		**Dominant Leg**	**Non-Dominant Leg**	
0 °/s		3.71 ± 0.87	3.78 ± 0.93	0.313	1.71 ± 0.37	1.61 ± 0.44	0.070
60 °/s		2.63 ± 0.45	2.46 ± 0.27	0.026	1.56 ± 0.26	1.51 ± 0.33	0.209
180 °/s		1.89 ± 0.30	1.83 ± 0.27	0.111	1.08 ± 0.22	1.07 ± 0.19	0.643

RIT—relative isometric torque; RPT—relative isokinetic torque; ACLR—anterior cruciate ligament reconstruction; SPLNV—supervised physiotherapy with a lower number of visits; SPHNV—supervised physiotherapy with a higher number of visits; *x*—mean; SD—standard deviation; *p* *—paired two-sample *t*-test with Bonferroni correction; *p* **—unpaired two-sample *t*-test with Bonferroni correction; *p* ***—unpaired two-sample *t*-test.

**Table 5:** Inter- and intragroup comparison between groups I SPHNV, II SPLNV and III CONTROL of relative isometric torque (RIT) obtained under isometric tension (0 °/s) and relative peak torque (RPT) values in the isokinetic conditions (60 °/s and 180 °/s) during I and II; measurements of extensor and flexor muscles of the knee joints were taken.

***KNEE JOINT EXTENSORS***					***p*** -value * **Group × Time**	***p*** **-value** **Group**	***p*** **-value** **Time**
**Angular Velocity**	**Time** **(Weeks after ACLR)**	**Group**					
		**Group I** ***x* ± SD**	**Group II** ***x* ± SD**	**Group III** ***x* ± SD**			
0 °/s	13	2.36 ± 0.79	1.95 ± 0.75	3.78 ± 0.93 ^b,c^	≤0.001	≤0.001	≤0.001
	24	3.72 ± 0.95 ^a^	3.19 ± 0.78 ^a^	3.71 ± 0.88			
	**∆**	1.36 ± 0.89	1.24 ± 0.87	−0.07 ± 0.29 ^b,c^	0.001		
60 °/s	18	1.97 ± 0.44	1.82 ± 0.41	2.46 ± 0.47 ^bc^	0.011	≤0.001	≤0.001
	24	2.56 ± 0.56 ^a^	2.25 ± 0.44 ^a^	2.63 ± 0.45			
	**∆**	0.60 ± 0.56	0.43 ± 0.43	0.17 ± 0.31 ^b^	≤0.011		
180 °/s	18	1.39 ± 0.37	1.34 ± 0.28	1.83 ± 0.27 ^b,c^	≤0.001	≤0.001	≤0.001
	24	1.81 ± 0.40 ^a^	1.65 ± 0.31 ^a^	1.89 ± 0.30			
	**∆**	0.42 ± 0.39	0.31 ± 0.26	0.06 ± 0.17^bc^	0.001		
***KNEE JOINT FLEXORS***					***p*** -value * **Group × Time**	***p*** **-value** **Group**	***p*** **-value** **Time**
**Angular Velocity**	**Time** **(Week after ACLR)**	**Group I** ***x* ± SD**	**Group II** ***x* ± SD**	**Group III** ***x* ± SD**			
0 °/s	13	1.09 ± 0.36	1.07 ± 0.21	1.61 ± 0.44 ^b,c^	0.010	≤0.001	≤0.001
	24	1.51 ± 0.38 ^a^	1.40 ± 0.42 ^a^	1.71 ± 0.37			
	**∆**	0.42 ± 0.34	0.33 ± 0.39	0.10 ± 0.24 ^b^	0.010		
60 °/s	18	1.40 ± 0.30	1.36 ± 0.31	1.51 ± 0.32	0.13	0.56	≤0.001
	24	1.55 ± 0.30 ^a^	1.51 ± 0.35 ^a^	1.56 ± 0.26			
	**∆**	0.16 ± 0.19	0.15 ± 0.22	0.04 ± 0.15	0.13		
180 °/s	18	0.98 ± 0.26	0.98 ± 0.23	1.07 ± 0.19	0.002	0.95	≤0.001
	24	1.16 ± 0.24 ^a^	1.13 ± 0.26 ^a^	1.08 ± 0.23			
	**∆**	0.18 ± 0.15	0.15 ± 0.16	0.01 ± 0.14 ^b,c^	0.002		

ACLR—anterior cruciate ligament reconstruction; *x*—mean; SD—standard deviation; ∆—difference between the results of subsequent measurements (result obtained at 24 weeks minus the result obtained at 13 weeks or 18 weeks). The comparison was performed using repeated measures ANOVA with post-hoc test (Tukey’s HSD test). The comparison of difference result between groups was performed using one way ANOVA with post-hoc test (Tukey’s HSD test). ^a^—statistically significant differences in the intra-group comparison (Tukey’s HSD test); ^b^—statistically significant differences between groups I and III (Tukey’s HSD test); ^c^—statistically significant differences between groups II and III (Tukey’s HSD test).

**Table 6:** Correlation between the number of postoperative physiotherapy visits and the obtained values of the relative isometric torque (RIT) during isometric tension (0 °/s) and relative peak torque (RPT) in the isokinetic tests (60 °/s and 180 °/s) in group I and separately in group II. The significance level of statistical differences (*p*); Pearson’s linear correlation coefficient (*r*).

**Test Correlation in Group I (*n* = 20)**						
**Group I** **(SPHNV)**	**Angular Velocity**		**Knee Joint Extensors**		**Knee Joint Flexors**	
			**Operated Leg**	**Uninvolved** **Leg**	**Operated Leg**	**Uninvolved** **Leg**
	0 °/s	***r***	0.194	0.222	0.289	−0.179
		***p***	0.412	0.346	0.217	0.450
	60 °/s	***r***	0.506	0.521	0.445	0.436
		***p***	0.023	0.018	0.049	0.054
	180 °/s	***r***	0.515	0.566	0.535	0.519
		***p***	0.020	0.009	0.015	0.019
**Test Correlation in Group II (*n* = 20)**						
**Group II** **(SPLNV)**	**Angular Velocity**		**Knee Joint Extensors**		**Knee Joint Flexors**	
			**Operated Leg**	**Uninvolved** **Leg**	**Operated Leg**	**Uninvolved** **Leg**
	0 °/s	***r***	0.305	0.091	0.048	−0.198
		***p***	0.191	0.704	0.839	0.402
	60 °/s	***r***	0.346	0.091	0.104	0.090
		***p***	0.135	0.703	0.662	0.707
	180 °/s	***r***	0.215	0.004	0.031	0.132
		***p***	0.363	0.988	0.897	0.579

## Data Availability

These studies were purely retrospective and observational studies.
